# Stem vacuole-targetted sucrose isomerase enhances sugar content in sorghum

**DOI:** 10.1186/s13068-021-01907-z

**Published:** 2021-03-01

**Authors:** Guoquan Liu, Yan Zhang, Hao Gong, Shan Li, Yunrong Pan, Christopher Davis, Hai-Chun Jing, Luguang Wu, Ian D. Godwin

**Affiliations:** 1 Centre for Crop Science, Queensland Alliance for Agriculture and Food Innovation, The University of Queensland, Brisbane, 4072 Queensland Australia; 2 School of Agriculture and Food Sciences, The University of Queensland, Brisbane, 4072 Queensland Australia; 3 Key Laboratory of Plant Resources, Institute of Botany, Chinese Academy of Sciences, Beijing, 100093 China

**Keywords:** Isomaltulose, Sorghum, Sucrose isomerase, Sugar content, Renewable energy, Photosynthesis, Genetic engineering, Sugarcane

## Abstract

**Background:**

Sugar content is critically important in determining sugar crop productivity. However, improvement in sugar content has been stagnant among sugar crops for decades. Sorghum, especially sweet sorghum with high biomass, shown great potential for biofuel, has lower sugar content than sugarcane. To enhance sugar content, the sucrose isomerase (*SI*) gene, driven by stem-specific promoters (*A2* or *LSG*) with a vacuole-targetted signal peptide, was transformed into the sorghum inbred line (T×430).

**Results:**

The study demonstrated that transgenic lines of grain sorghum, containing 50–60% isomaltulose, accumulated up to eightfold (1000 mM) more total sugar than the control T×430 did (118 mM) in stalks of T_0_ generation. Subsequently, the elite engineered lines (A5, and LSG9) were crossed with sweet sorghum (Rio, and R9188). Total sugar contents (over 750 mM), were notably higher in F_1_, and F_2_ progenies than the control Rio (480 mM). The sugar contents of the engineered lines (over 750 mM), including T_0_, T_1_, F_1_, and F_2_, are surprisingly higher than that of the field-grown sugarcane (normal range 600–700 mmol/L). Additionally, analysis of physiological characterization demonstrated that the superior progenies had notably higher rates of photosynthesis, sucrose transportation, and sink strength than the controls.

**Conclusions:**

The genetic engineering approach has dramatically enhanced total sugar content in grain sorghum (T_0_, and T_1_) and hybrid sorghum (F_1_, and F_2_), demonstrating that sorghum can accumulate as high or higher sugar content than sugarcane. This research illustrates that the *SI* gene has enormous potential on improvement of sugar content in sorghum, particularly in hybirds and sweet sorghum. The substantial increase on sugar content would lead to significant financial benefits for industrial utilization. This study could have a substantial impact on renewable bioenergy. More importantly, our results demonstrated that the phenotype of high sugar content is inheritable and shed light on improvement for other sugar crops.

**Supplementary Information:**

The online version contains supplementary material available at 10.1186/s13068-021-01907-z.

## Background

Sugar yield, largely impacted by biomass and sugar content, is a key determinant of economic sustainability for sugar crops. In recent decades, improvement on sugar yield has been achieved almost entirely by increasing biomass [1–3], despite the higher commercial value and higher heritability of sugar content [4]. Recent studies on manipulation of plant genes, which are involved in sugar metabolism, have been unsuccessful for increasing sugar content in sugar crops [5–7]. There is significant pathway redundancy in elite cultivars to buffer against increases on sucrose levels by manipulating a single gene [8]. Multiple mechanisms appear to contribute to the upper limit of sugar concentration, including regulation in signal transduction from specific (e.g., sucrose) or broad (e.g., osmotic) sensors, thermodynamic limitations (e.g., leakage of sucrose through storage compartment membranes), or energetic limitations (e.g., continuous ‘futile’ cycle of sucrose cleavage and synthesis within the storage pool) [9–12].

Among sugar crops, sugarcane accounts for almost 80% of global sugar production. Sweet sorghum has displayed huge potential to be multiple sources of energy, food and animal feed and could be a substitute for sugarcane to produce biofuel [13, 14]. It grows quickly in adverse stress conditions of marginal lands in tropical, subtropical and temperate zones. It is a C_4_, drought tolerant, high biomass, and high water use efficiency crop that produces stalks up to five meters tall, accumulating sucrose (α-D-glucopyranosyl-1,2-D-fructofuranose). However, current sweet sorghum varieties, producing comparatively low sugar content (around 500 mmol/L), urgently require plant breeders to improve sugar accumulation in stalks for biofuel [13].

Sucrose can be converted into isomaltulose (α-D-glucopyranosyl-1,6-D-fructofuranose) by certain bacteria [15]. Unlike sucrose, isomaltulose cannot be digested by invertases [16] nor be metabolized by majority microbes, including the predominant oral microflora, presenting benefit in many foods as an acariogenic sweetener [17]. Meanwhile, isomaltulose can be digested by humans with the same glucose/fructose as primary products and have the same final energy value as sucrose. Interestingly, the first step of digestion involves an intestinal disaccharidase rather than salivary invertase, which slows down the isomaltulose digestion. The slow process of digestion results in less fluctuation of glucose and insulin concentration in blood [18]. Therefore, isomaltulose has a growing demand as a stable, slowly digestible, acariogenic, non-hygroscopic sugar in the modern world [18–20]. Futhermore, isomaltulose has an accessible carbonyl group, which makes it attractive as a renewable starting material for manufacture [21]. The application is currently limited due to the high cost of isomaltulose production through fermentation [22, 23].

Isomaltulose can be produced through expression of the sucrose isomerase (*SI*) gene in plants [24]. Isomaltulose, compared to sucrose, is very slowly metabolized and cannot be transported in plants [25], hence the site of isomaltulose production becomes a storage. Exogenous application of isomaltulose triggers some plant sugar sensing mechanisms and changes gene expression profiles differently from sucrose [25, 26]. Previously, it was demonstrated that the efficient conversion of sucrose into the non-metabolized isomer (palatinose) is disruptive or lethal for plant development [27]. The tuber-specific expression of the apoplasm-targeted *SI* allowed the partial conversion of sucrose to isomaltulose in potato, but the total non-structural carbohydrate content was decreased [28, 29]. Significant progress has been made in last two decades. Recent reports have indicated that the N-terminal pro-peptide (NTPP) fragment from sweet potato sporamin can deliver various proteins to the sugarcane vacuole, but low pH and high protease activity make the vacuole environment hostile [30]. With the availability of strong stem-specific promoters, a highly efficient *SI* gene, and silencing motifs, high concentration of isomaltulose (up to 483 mM or 81% of total sugars) has been successfully achieved in sugarcane [15, 24, 31]. To our best knowledge, similar investigations have not been reported in other biomass species yet.

In the storage parenchyma cells of mature stems of sweet sorghum, the sugar storage vacuole occupies about 90% of the symplast and 80% of the total tissue space. The vacuole stores a correspondingly large proportion of sucrose, which can accumulate up to 500 mM in stem juice. Our objective was to improve sugar content by targeting stem vacuole with the *SI* gene. We hypothesized that high isomaltulose concentration could be accumulated in stalks of engineered lines and lead to high total sugar content in sorghum, especially sweet sorghum. Considering the existing transformation system on grain sorghum and the recalcitrance on sweet sorghum transformation, we strategically avoid transforming sweet sorghum directly in this project [32, 33]. However, investigation on grain sorghum and hybrids (sweet × grain sorghum) could provide insightful information on sugar accumulation in commercial hybrids and sweet sorghum.

## Results

### Isomaltulose was efficiently accumulated in T_0_ transgenic lines

**Fig. 1:**
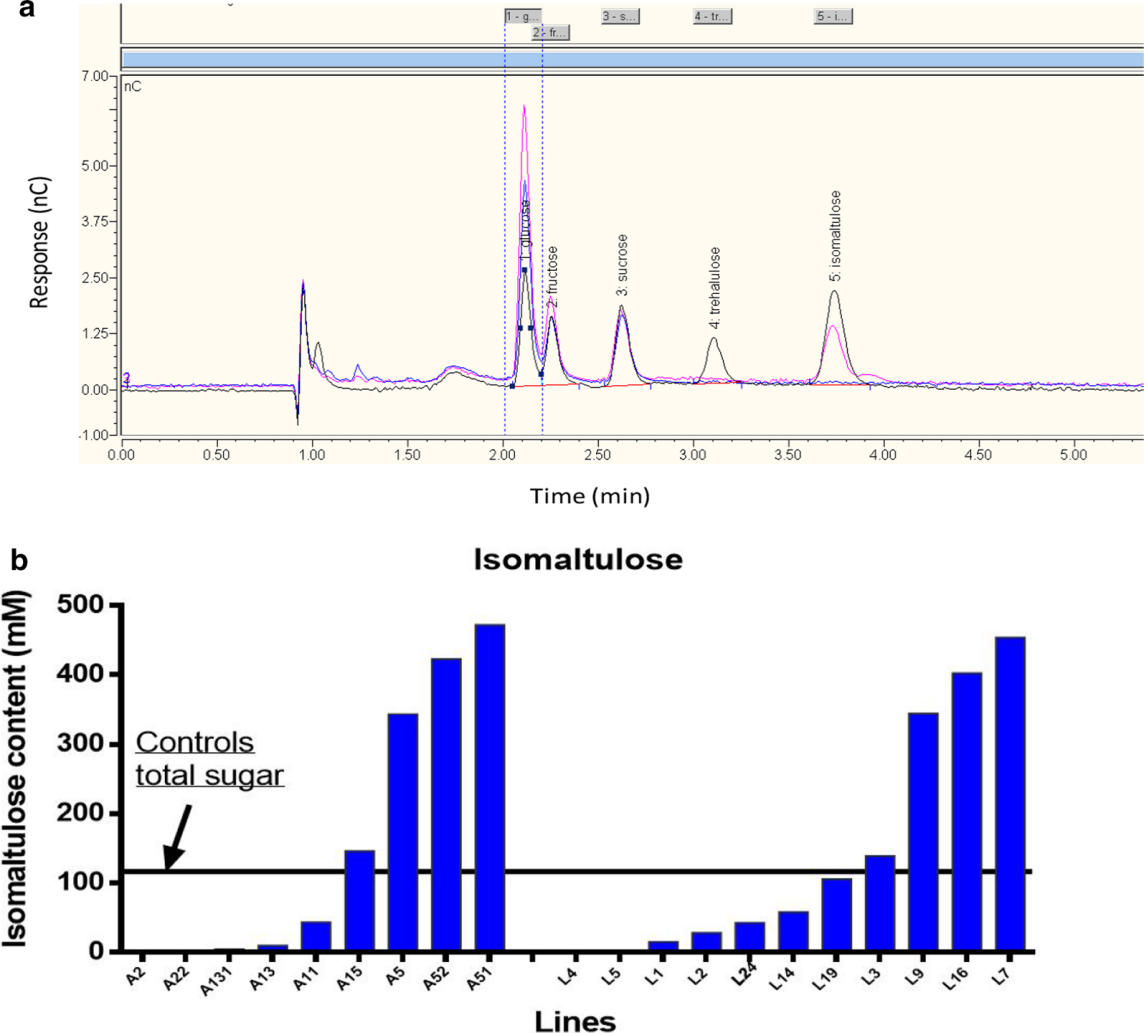
Screening transgenic T×430 sorghum lines for the presence of isomaltulose (IM) in stem juice. **a** High-performance liquid chromatography (HPLC) profiles. Black curve: Standard solutions contained glucose, fructose, sucrose, trehalulose and IM; Pink curve: Diluted (16,000x) juice from transgenic sorghum stalk internode 4 showing isomaltulose (last peak #5) was accumulated beyond glucose, fructose and sucrose; Blue curve: Diluted (16,000x) juice from a parent control sorghum stalk showing no isomaltulose accumulated. **b** isomaltulose concentrations in juice from the internode 4 of the transgenic lines. The line’s label starts with A driven by *A1* promoter and with L driven by *LSG2* promoter. The plants were the first vegetative generation from tissue culture with around 7 internodes when sampled 20-day post-anthesis. A horizontal line was drawn on the highest total sugar content (sucrose equivalent) among the five T×430 plants (Controls)

Twenty independent transgenic lines were demonstrated to contain the *sucrose isomerase* (*SI*) gene using the polymerase chain reaction (PCR) analysis. Among these lines, 16 displayed detectable isomaltulose levels by high-performance liquid chromatography (HPLC) in stalks (Fig. 1a). Up to 446 mM isomaltulose was accumulated in stalk juice, which was fourfold higher than the total sugar content of the untransformed T×430. The isomaltulose concentrations were substantially variable among lines (Fig. 1b). Similar patterns were observed in two transgenic populations driven by different promoters of *A1* or *LSG2* (Fig. 1b).

Because the UQ68J *SI* gene is highly specific for producing isomaltulose [24], trehalulose concentrations were generally below 5% of the isomaltulose concentrations in the corresponding internodes (Additional file 1: Table S1). The majority of transgenic lines were morphologically similar and equivalent to the untransformed control T×430 in the glasshouse (Additional file 1: Fig. S1). Transgenic plants flowered at a similar time as the control T×430 (Additional file 1: Fig. S1).

The roots and leaves were tested from all the transgenic lines, isomaltulose concentrations were below 5 mM in roots. Isomaltulose concentration increased with age in leaves to a maximum of about 20 mM, which is consistent with the expression patterns for the ‘stem-dominant’ promoters [34, 35]. However, *SI* enzyme activity could not be detected from cell extracts of transgenic roots or leaves. The negative effect on sorghum growth was not observed due to the small amount of isomaltulose accumulation in roots and leafs (Additional file 1: Fig. S1). Despite substantial isomaltulose accumulation in stalks, SI enzyme activity was below the detection threshold in cell extracts, indicating a short half-life of this protein after delivery into the acidic/proteolytic sucrose storage vacuoles.

### Total sugar content was greatly enhanced in T_0_ transgenic lines

**Fig. 2:**
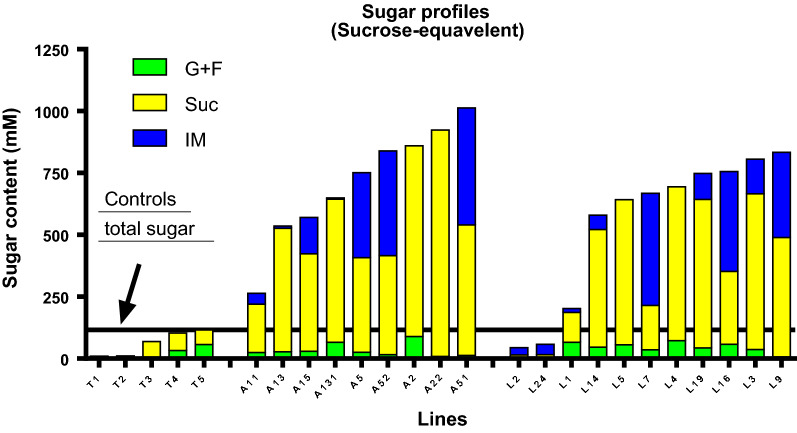
Total sugar profile of the internode 4 in controls T×430 and transgenic lines. A horizontal line was drawn on the highest sugar content of the control T×430. G + F: ½ (Glucose plus fructose); *Suc* Sucrose, *IM* Isomaltulose. T1 to T5: five untransformed T×430 samples; The line’s label starts with A driven by *A1* promoter and with L driven by *LSG2* promoter

The total sugar content has been notably increased in 20 T_0_ transgenic lines compared to the wild-type control except two lines (L2, and L24), regardless of which promoter used (*A1* or *LSG2*) (Fig. 2). The total sugar contents in internode number 4 of most lines were in a range of 600 to 1,000 mM, which was equivalent to five to eight folds of the control (Additional file 1: Table S1). These concentrations were comparable or even higher than that of the field-grown sugarcane (normally 600–700 mM). The predominant components of sugar were sucrose and isomaltulose in transgenic lines; meanwhile, their glucose and fructose contents were similar to the parent (Fig. 2). Unexpectedly, some transgenic lines such as L4 and A2 had no detectable isomaltulose but sucrose contents were enhanced fivefold to eightfold when compared to the control T×430 (Fig. 2), regardless of the promoter used.

### High sugar contents were accumulated across internodes of elite transgenic stalks

**Fig. 3:**
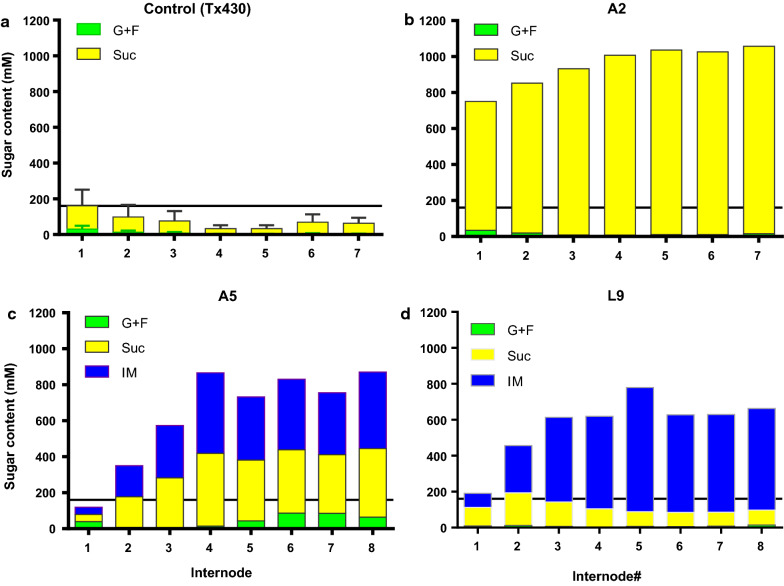
Sugar profile of internodes in controls T×430 and transgenic lines. The plants were sampled 20-day post-anthesis with 7–8 internodes. G + F: ½ (Glucose plus fructose); Suc: Sucrose; IM: Isomaltulose. Results from the T×430 controls are means of five replicates, with standard errors. A horizontal line on each panel was drawn on the highest sugar content of internode 1 of the control T×430. **a** The controls T×430; **b** Transgenic line A2; **c** Transgenic line A5; and **d** Transgenic line L9

Three transgenic lines, designated A2, A5 (both driven by *A1* promoter) and L9 (driven by *LSG2* promoter), with high-sugar content were selected for further characterization on sugar profiles in developmental stages. Lines A5 and L9 accumulated high levels of isomaltulose up to 691 mM in juice from mature internodes (Fig. 3c, d). Compared to the control T×430, the transgenic lines with high yields of isomaltulose did not show commensurable reduction but enhanced sucrose content in most internodes (Fig. 2). Surprisingly, isomaltulose could not be detected in any A2 tissues including all internodes of the stalks, but sucrose content accumulated eightfold higher than the level in the control T×430 (Fig. 3b).

Further investigation on T_1_ progenies of A2, A5, and L9 has been performed and focused on heritability of high sugar content. Up to twelve samples of each progeny have been analyzed. T_1_ progenies of L9 outperformed the counterparts of A2, and A5 in terms of high heritability. Because no isomaltulose was detected in A2, the phenotype of high sugar content did not transmit to the next generation. T_1_ progenies of A5 accumulated up to 367 mM isomaltulose and up to 550 mM total sugar content, those sugar contents in T_1_ progenies were not as high as sugar contents in T_0_ generation. The results of L9 T_1_ progeny samples were very promising and displayed high heritability of high sugar content (up to 896 mM in stalk). Positive samples have accumulated much higher sugar content than null-segregants (nil-samples) and the wild-type controls (Additional file 1: Fig. S4).

### Real-time PCR was performed on A5, L2, and L9 T_1_ generation

**Fig. 4:**
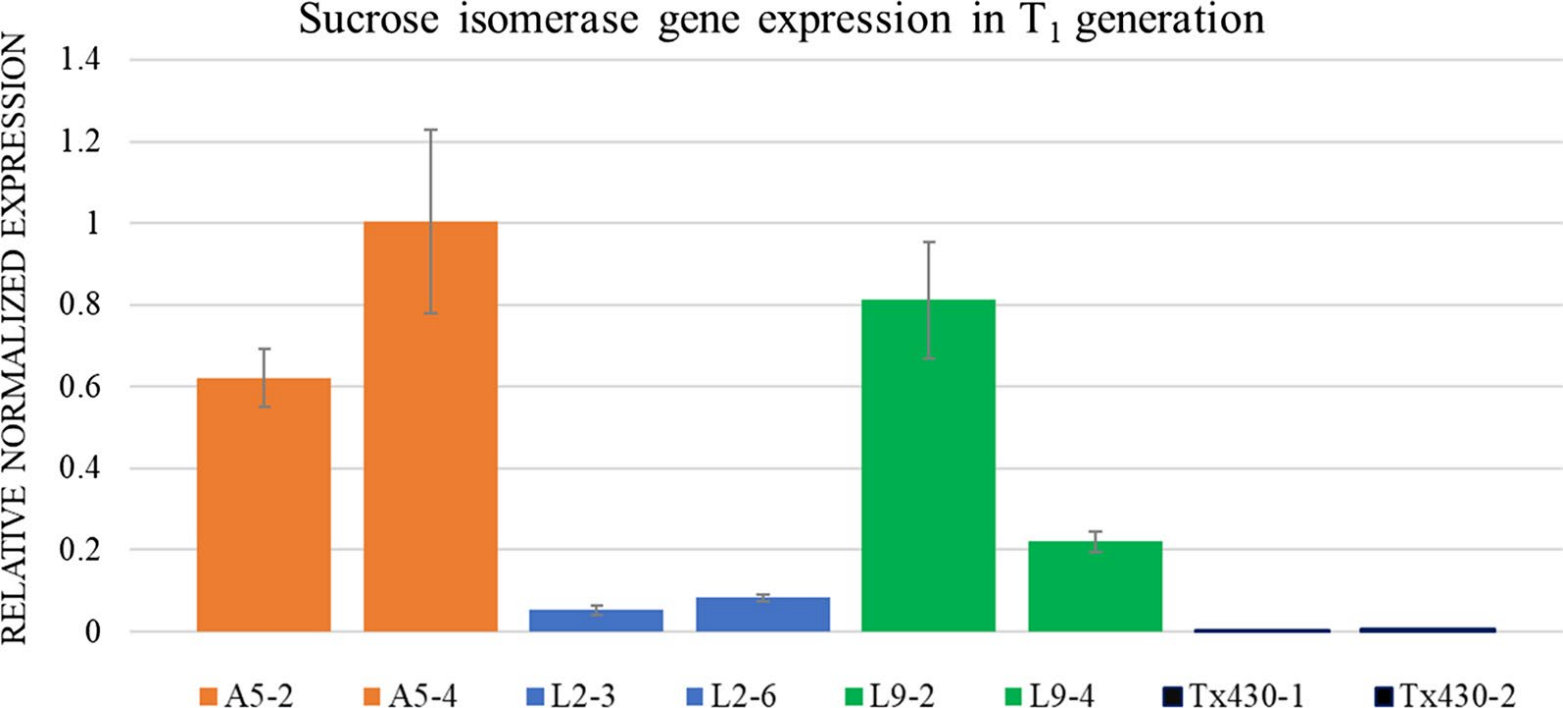
RT-PCR analysis of the *SI* gene expression in T_1_ progenies and T×430. Six *SI*-positive T_1_ lines are from three T_0_ transgenic events. A5 T_1_ progenies: A5-2 and A5-4; L2 T_1_ progenies: L2-3 and L2-6; L9 T_1_ progenies: L9-2, and L9-4; T×430-1, T×430-2 are non-transgenic control samples. The error bars presented the variation in three biological replicates

Quantitative real-time PCR was deployed to determine the *SI* gene expression in different transgenic lines. The elite transgenic lines, accumulating high isomaltulose, and high total sugar, A5 and L9 were selected. Line L2, with poor isomaltulose accumulation, was chosen for comparison. Non-transgenic T×430 was used as the wild-type control. T_1_ Positive progenies of A5, L2, and L9 were identified by PCR screening of the *SI* gene (Additional file 1: Fig. S5). The RT-PCR results revealed that A5 and L9 displayed a relatively high levels of *SI* gene expression, which are in agreement with their high levels of isomaltulose accumulation. L2 showed comparatively low level of *SI* gene expression, which aligned with poor isomaltulose accumulation. As expected, no *SI* gene expression was detected in stalks of the wild-type T×430 (Fig. 4).

### High sugar contents were inherited in F_1_ hybrids

The elite sweet sorghum cultivar R9188, and Rio were selected as female lines for crossing due to their advantages of large biomass and high-sucrose content in stalks. Transgenic lines A5, and L9 were chosen as male lines because of their superior performance on isomaltulose accumulation and high total sugar content. Crosses were performed with the male-sterile lines of R9188, and Rio. Hybrid seeds were harvested from successful crossing.

**Fig. 5:**
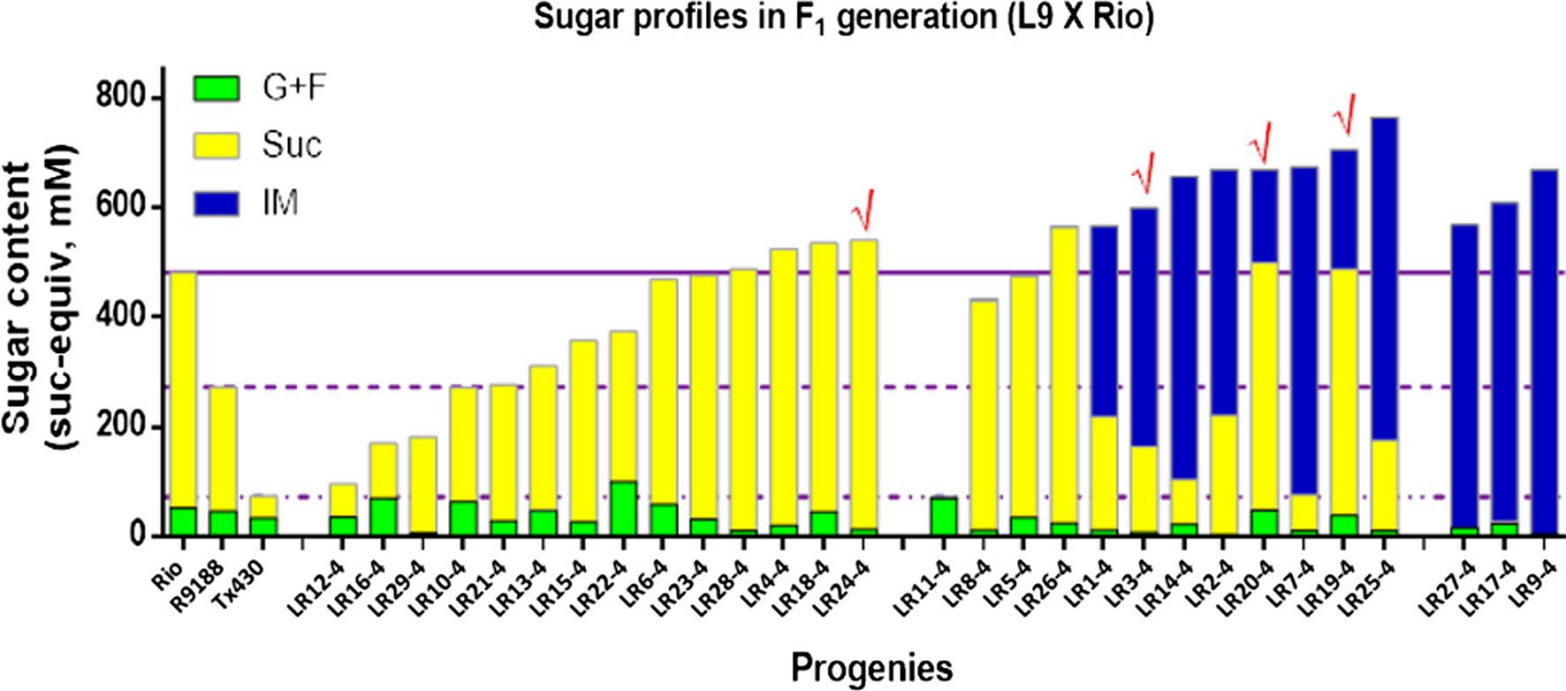
Total sugar content in the F_1_ hybrids (Rio X L9). Sugars were measured 20-day post-anthesis in the middle section of internode 4 (counted from top). L9 is the transgenic line driven by *LSG2* promoter. G + F: ½ (Glucose plus fructose); Suc: Sucrose; IM: Isomaltulose. Bars of the controls Rio, R9188, and T×430, were means of three stalks. Three horizontal lines represent the average of the three controls, respectively. The progenies with red ticks (√) were selected for further testing

Thirty seeds of hybrids of Rio X L9 were sown in pots along with the controls of Rio, R9188, and T×430 in the glasshouse. The sweet sorghum cultivar R9188 is another version of Rio with an extra dwarf gene, hence almost 50 cm shorter. Hybrid seed germination and early seedling growth were similar to the controls, except one hybrid seed which did not germinate. Sugar profiles of HPLC showed that among 29 progenies of F_1_ generation, isomaltulose was detected in 15 progenies (51.7%) and no isomaltulose was detected in the rest of 14 samples (48.3%). The ratio of positive to negative samples was close to the predicted 1:1 ratio (Fig. 5), indicating hybrid seeds inherited the *SI* gene as a single genetic locus from the parent L9.

Within the 15 isomaltulose positive group, three progenies converted almost all sucrose into isomaltulose; six converted more than 65% of sucrose; two converted about 33% of sucrose; four had less than 1% sucrose converted (Fig. 5). Notably, the improvement of total sugar content was observed in most isomaltulose positive lines (Fig. 5). The increase of total sugar content was on average 37% higher than the sweet sorghum Rio. The increase ranged from 484 to 932% if compared with the grain sorghum T×430, which is in agreement with the results of the T_0_ generation (Fig. 2).

Another hybrid population of R9188 X L9 were planted as well. It showed similar pattern as the population of Rio X L9. Among 26 F_1_ population, 12 of them are positive for sucrose isomerase gene (Additional file 1: Table S2). The highest total sugar content at 764 mM was measured in F_1_ L9R9-20 line and the top isomaltulose content at 565 mM was detected in the F_1_ L9R9-9 line. By comparison, remarkably higher total sugar contents were monitored in positive *SI* lines (on average 538 mM) than negative *SI* lines (on average 342), which means the sugar content has been improved 57.3% thanks to the *SI* gene. While the average sugar content in the sweet sorghum R9188 and grain T×430 were 261 and 93 mM, respectively. The detail of results was shown in Additional file 1: Table S2.

### High sugar contents were inherited in F_2_ population

Based on isomaltulose concentration, total sugar content, stalk biomass, and seed production, F_1_ (Rio X L9) progenies LR3, LR19 and LR20 were selected for further characterization. With the parental controls of sweet sorghum Rio, progeny LR24, a null segregant with comparative high sugar content was also selected as a hybrid control. Seeds were produced by self-pollination.

**Fig. 6:**
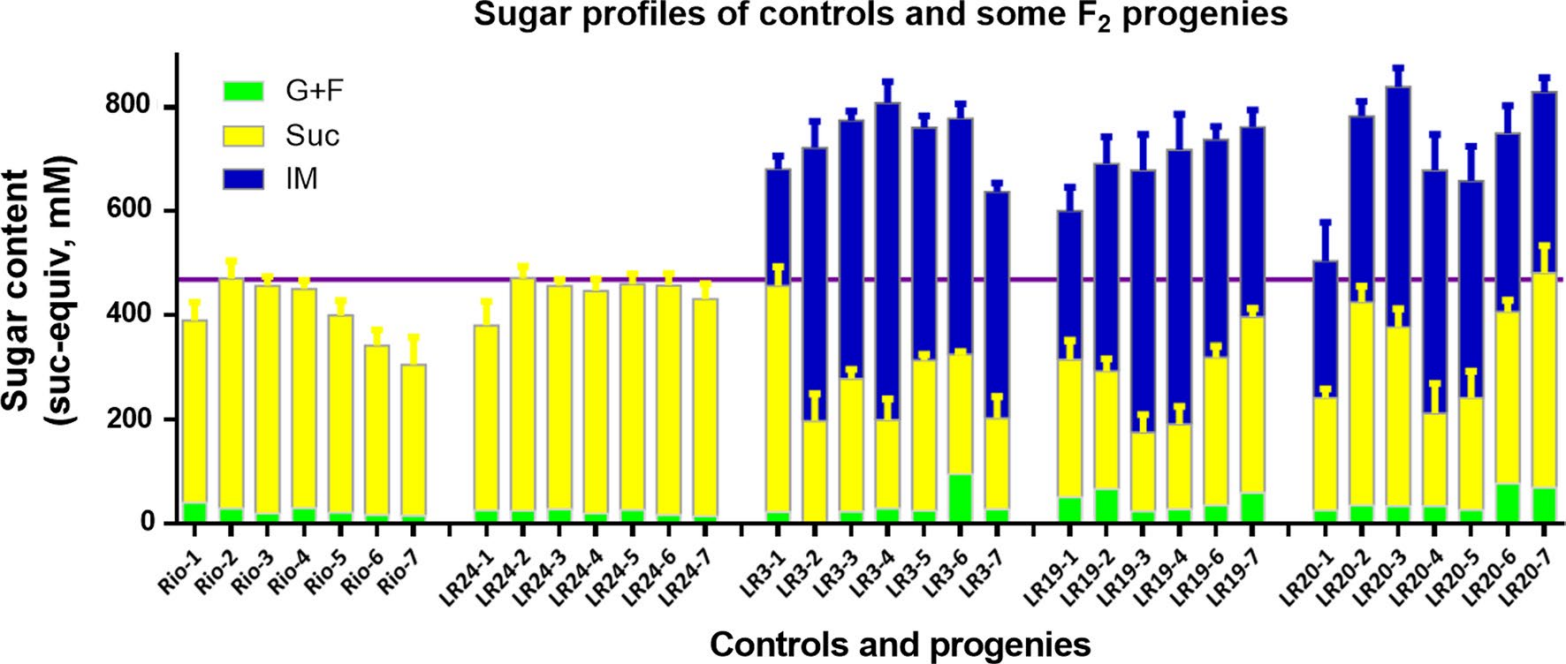
Sugar profile of internodes in controls and selected progenies of the F_2_ generation (Rio X L9). The first group (Rio-1 to 7) represents the parent Rio control, the second group LR24 (LR24-1 to 7) represents the transgene negative control F_2_ progenies, and the rest three groups are PCR positive F_2_ progenies from LR3, LR19 and LR20. The last digit in the label of the X-axis is the internode number counted from the top. G + F: ½ (Glucose plus fructose); Suc: Sucrose; IM: Isomaltulose. Sugars were measured 20-day post-anthesis in the middle section of each internode. Results were means with standard errors from three replicates. The horizontal line was drawn on the highest total sugar content among all internodes of the Rio control

Sugar profiles of the isomaltulose positive plants showed that they inherited the phenotype of high isomaltulose and high sugar content (Fig. 6). In all *SI* positive progenies, isomaltulose accumulated at high levels in all internodes. In addition, sucrose content were stored at comparable levels (total sugar content up to 812.2 mM), resulting in up to 69% increase of total sugar content compared to the parental (480.6 mM) or the hybrid control (470.9 mM) (Fig. 6).

### Sugar content was increased, whereas water content was decreased in F_2_ stalk juice

**Fig. 7:**
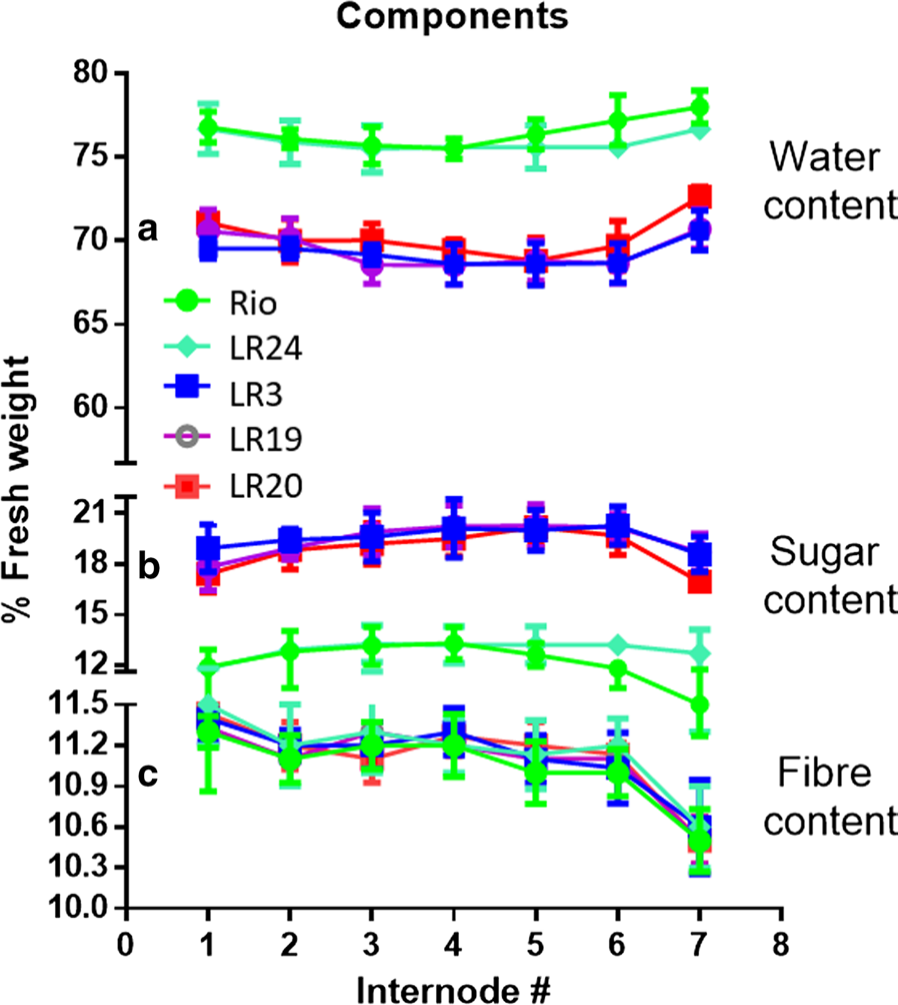
Total water (**a**), sugar (**b**), and fiber (**c**) contents in the internodes of controls and the F_2_ progenies. Rio was the parental control, LR24 was transgene negative F_2_ progeny as the hybrid control. LR3, LR19, and LR20 were transgene F_2_ positive progenies. Internodes were numbered from the top. Results were means with standard errors from three replicate plants

Carbon partitioning into sugars and fiber was estimated in the selected F_2_ progenies and controls. There was more sugar per unit fresh weight (FW) in all internodes of the tested high-sugar progenies than the controls (Fig. 7a). In the sweet sorghum Rio and hybrid null segregant LR24, the water content was typically constant around 75% along the stalk with a slight increase in the bottom internodes; however, in the stalks of three high-sugar progenies, water content was significantly lower around 70% (Fig. 7b). Moreover, there were no significant change in the fiber content among all samples, which was around 11% in internode tissues (Fig. 7). These results indicated that instead of alteration of fiber and sugar, assimilation was improved and more sugar was stored in the progenies LR3, LR19, and LR20 than the controls. Therefore, the commercially important trait of high sugar content in juice from the selected progenies are underpinned by increasing sugar content and decreasing water content in the mature stalk.

### Photosynthesis was increased in high sugar F_2_ lines

**Fig. 8:**
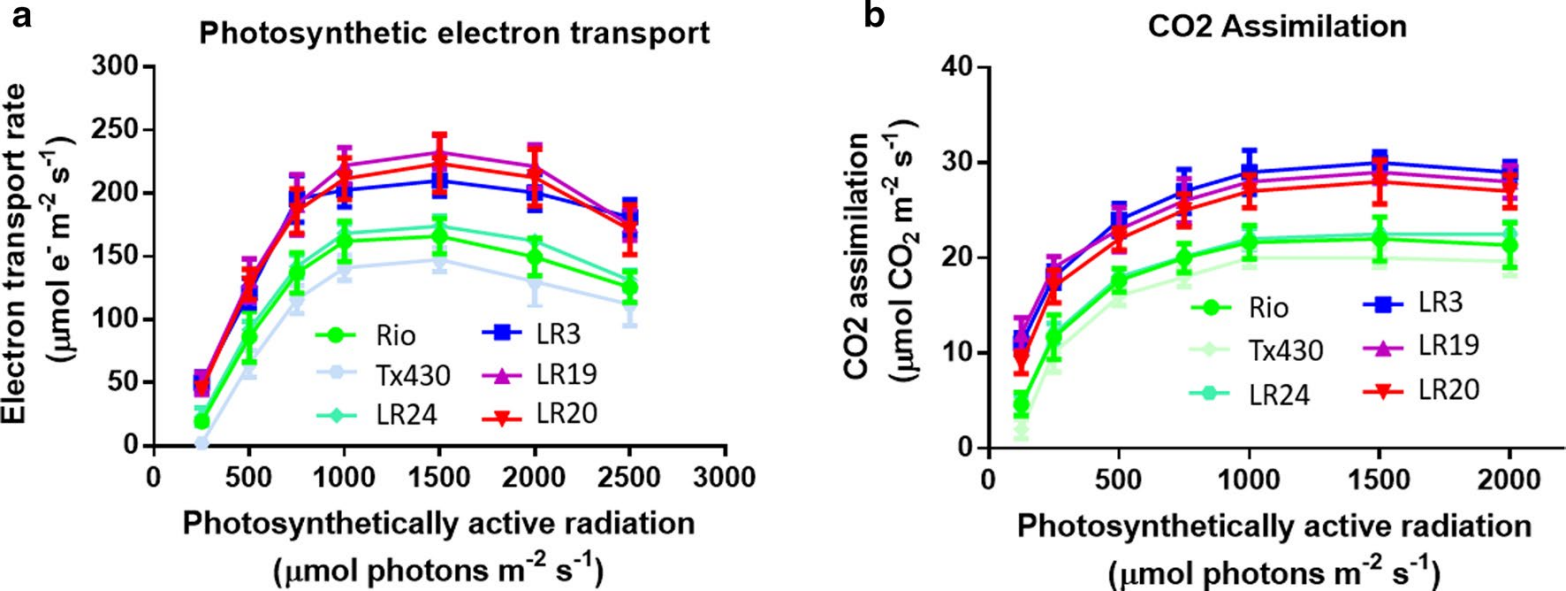
Photosynthetic electron transport rate (**a**) and CO_2_ assimilation (**b**) in controls and the F_2_ progenies. Three controls: Rio (parental control), T×430 (untransformed control), LR24 was transgene negative F_2_ progeny as the hybrid control. Three high-sugar transgene F_2_ positive progenies: LR3, LR19, and LR20. Photosynthesis was measured in the second leaf from the top after 10–11-day post-anthesis. Results were means with standard errors from three replicates

Two key physiological characteristics, including photosynthetic electron transport and CO_2_ assimilation, were examined to understand the mechanisms of enhanced sugar accumulation. Rates of leaf electron transport and CO_2_ assimilation of the progenies LR3, LR19, and LR20 were higher than the controls Rio, T×430 and hybrid control LR24. The photosynthetic electron transport rate and CO_2_ assimilation rate were measured by chlorophyll fluorescence (reflecting photosynthetic efficiency in photosystem II). The both rates were improved by 20% to 35% in high sugar content F_2_ lines compared to the controls at different photosynthetically active radiation (PAR) levels (Fig. 8). Light response curves from the fully expanded leaf 2 are measured (Fig. 8). Moreover, the senescence of the bottom leaves on each stalk of the high sugar progenies was typically delayed by 2–3 weeks, resulting in leaf functional extension in photosynthesis.

### Sugar transport was improved in source leaves and sink tissues

**Fig. 9:**
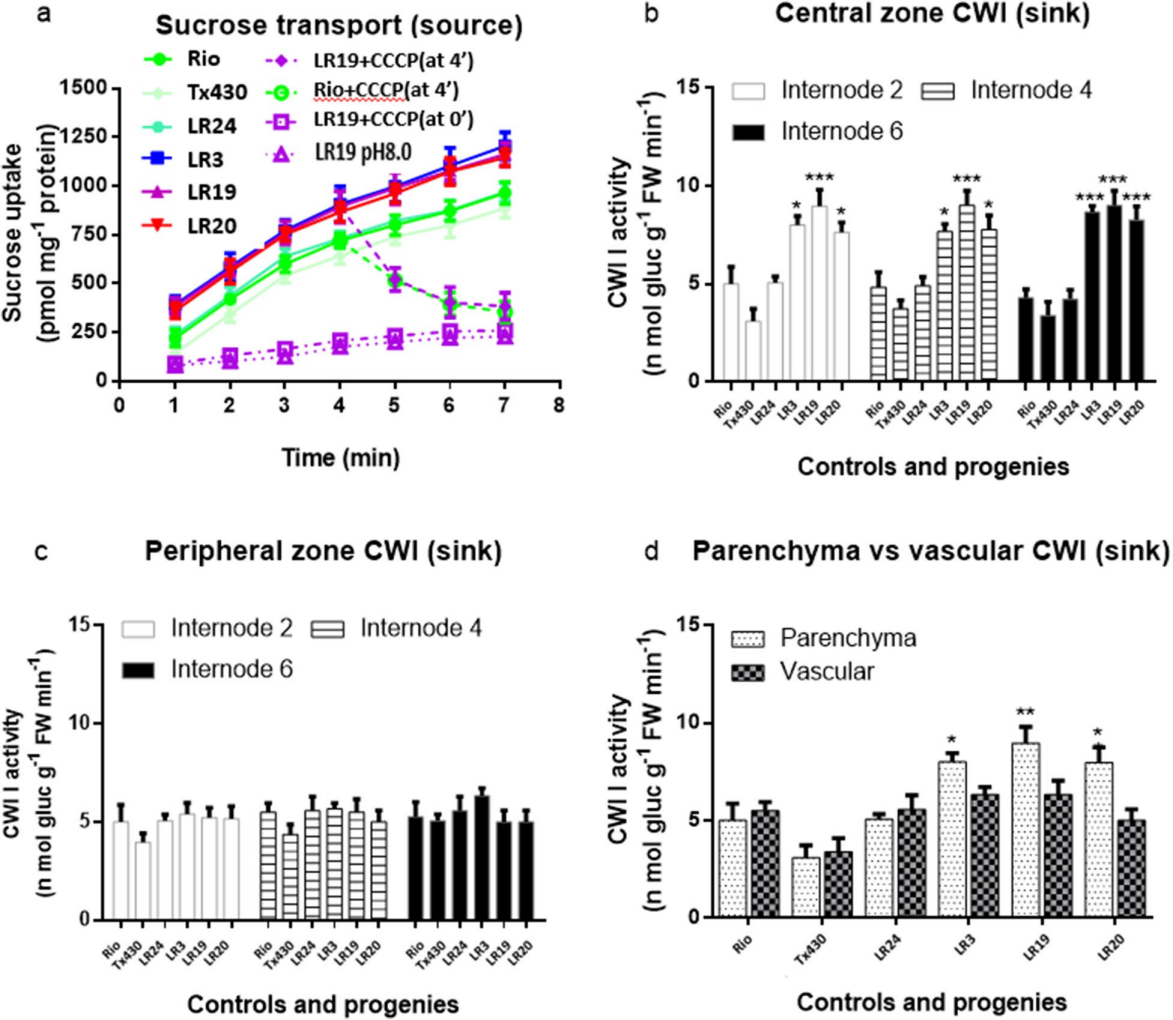
Relationship of source and sink in controls and the F_2_ progenies. **a** sucrose transport (source). **b** CWI activity was measured in the central parenchyma-rich zone (sink). **c** in the peripheral vascular-rich zone (sink). **d** in separated vascular bundles and parenchyma tissue from the central zone of internode 5 (sink). Three controls: Rio (parental control), T×430 (untransformed control), LR24 was transgene negative F_2_ progeny as the hybrid control. Three high-sugar transgene F_2_ positive progenies: LR3, LR19, and LR20. CCCP: carbonyl cyanide m-chlorophenyl hydrazone. The leaves and internodes were sampled at 20 days after anthesis. Results are means with standard errors from three replicates. Analysis of variance (ANOVA) with Bonferroni post-tests showed significant differences between any control and high-sugar progenies in the sucrose transport rates at all time points. The same statistical analysis showed significant differences between controls and high-sugar progenies in CWI activity of parenchyma cells in the central zone. *P < 0.05; **P < 0.01; ***P < 0.001

Rate of proton gradient-dependent sucrose transport into plasma membrane vesicles (PMV) is an indicator for sucrose uploading in the source leaves [36]. The isolated PMVs from leaf 2 and 3 of the selected high-sugar progenies LR3, LR19, and LR20 were 20% to 40% higher than that of controls (null segregant LR24, parents Rio and T×430), indicating the driving power of loading assimilation for transport was improved (Fig. 9a) in the source leaves of the high-sugar progenies.

Sorghum phloem in a stem vascular bundle is symplasmically isolated from the surrounding parenchyma cells, and the sucrose unloading is apoplasmic [37]. Cell wall invertase (CWI) activity is a determinant of sucrose gradient in the unloading area. In all tested internodes, CWI activities of the central storage parenchyma-rich zone were significantly higher in the high-sugar progenies than in the controls LR24, Rio and T×430 (Fig. 9b), but not in the peripheral vascular-rich zone (Fig. 9c). When the vascular bundles were dissected from the storage parenchyma cells in the central zone of internode 5 and assayed separately, the increased CWI activity in the high-sugar progenies was clearly restricted to the storage parenchyma (Fig. 9d), indicating the abilities on assimilate was increased within the sink tissues of the high-sugar progenies.

## Discussion

The present study demonstrated that notably higher sugar contents (over 750 mM) were achieved in transgenic grain sorghum (T_0_, and T_1_) and sweet x grain sorghum hybrids (F_1_, and F_2_), which is similar or higher than the sugar content of field-grown sugarcane (600–700 mM). The high sugar content, which was detected by HPLC in T_0_, T_1_, F_1_, and F_2_ plants, displayed that the phenotype of high level of sugar accumulation was stably inheritable. This study displayed that sucrose isomerase can efficiently convert sucrose into isomaltulose and dramatically increase total sugar content in sorghum. In addition, the superior engineered progenies had significantly higher photosynthesis, higher sucrose transport, and higher sink strength than the controls, which could be the key drivers for higher sugar accumulation in plants. This approach provides a new perspective on the plant source-sink relationship. It could have a substantial impact on producing high-value sugar isomaltulose and have enormous potential for renewable feedstcoks for bio-energy.

The current research on sorghum is beneficial from previous study on sugarcane. Firstly, sucrose depletion was avoided by targeting the *SI* enzymes into sucrose-storage vacuoles [38]. Secondly, the disturbance on normal growth/functions of other organs was circumvented using stem-specific expression of the *SI* gene [31, 35]. Finally, the *SI* gene sequence was modified to remove the motifs that trigger silencing in plants [31, 39]. To our best knowledge, this would be the first report on engineering *SI* in sorghum, or any other cereal. Sweet sorghum has been considered as a biofuel and biomass crop [13]. Our results displayed that sugar content can be increased up to 69% in hybrids compared with sweet sorghum, which will bfoost industrial value at large scale.

The activity of the vacuole-targeted SI enzyme was undetectable in cell extracts, because the sucrose-storage vacuoles are highly acidic and proteolytic. Rapid degradation of vacuole-targeted SI presumably protects against running out of sucrose in growing tissues. It is believed that isomaltulose accumulates gradually in the stalk during development, probably due to following reasons: (i) constant transcription of *SI* driven by the strong stem-specific *LSG2* or *ScR1MYB1 A1* promoter [31, 35]; (ii) high catalytic efficiency allowing occasional isomaltulose production before *SI* inactivation [24]; and (iii) very slow isomaltulose metabolism by plant enzymes [40]. For commercialization of this valued sugar, it is essential to achieve proper patterns of developmental expression, cell compartmentation, and enzyme stability to yield high isomaltulose content in stalks.

There has been an ongoing discussion as to whether current sugar crops have reached a physiological plateau with respect to sugar accumulation [41]. Compared to the sugar content of field-grown sugarcane juice (600–700 mM), high-level sugar accumulation (> 1000 mM disaccharides content), containing isomaltulose production (up to 691 mM) in stalk juice of the transgenic line in this study, sheds lights on that the assumed ‘ceiling’ above sugar accumulation could be exceeded.

Transgenic sorghum lines provide new insightful information on mechanisms as to how plants regulate sugar accumulation, a pivotal question in plant biology [42–45]. The phenotype of high total sugar content is attributed to delaying leaf senescence, increasing photosynthetic activity, and enhancing sucrose loading rates in source tissues, as well as higher activity in stalk storage parenchyma of CWI, which has multiple roles in sink tissues [44, 46]. Each of these activities would make a contribution to high sugar yield. Further comparative analysis of the superior lines and their parent lines could reveal key molecular and physiological control points in plant source-sink flux. As all the reported experiments were undertaken under well-watered, temperature-control glasshouse conditions, it is essential that further field trial should be undertaken, given the considerable diurnal and seasonal temperature variations, as well as water and nutrient availability.

Sweetness is an important commercial trait in many food crops. Enhancing sweetness through a slowly digested, acariogenic sugar, such as isomaltulose, can bring direct health benefits for consumers [18]. Isomaltulose is naturally present at a very low level (0.1–0.7%) in honey and sugarcane extracts which are too small to be extracted [18]. In this study, isomaltulose can be accumulated at a notably high level (691 mM) in transgenic sorghum lines. It could be harvested and extracted at the commercial scale in the future.

The fermentable carbohydrate content is also a key determinant of the economic and environmental feasibility of renewable biofuel production [47, 48]. Sweet sorghum is widely considered as a biofuel crop [1]. Accumulation of higher sugar content would increase the economic value of renewable energy. In the long term, sugars ultimately underpins all other biosyntheses in plants. The sugar boosting effect of the *SI* gene may be a foundation for higher sugar yields of many other bioenergy materials.

## Conclusion

Significant progress has not been made in sugar content among sugar crops for decades. The genetic engineering has shed lights on improving sugar content of sugar crops. The *SI* gene has been successfully transformed into sorghum and drastically improved total sugar content (up to 1000 mM) in sorghum. Generally, the total sugar content ranges 400 to 500 mM, 600 to 700 mM in stalk juice of sweet sorghum and sugarcane, respectively. Remarkably, the high sugar contents (more than 750 mM) were measured in multiple transgenic events (A5, A51, A52, L3, L7, L9 and L16) and multiple generations (T_0_, T_1_, F_1_, and F_2_). The total sugar content in F_1_ and F_2_ generations have been improved 57 and 69%, respectively, compared with the elite sweet sorghum Rio (close to 500 mM). Those total sugar contents of elite engineered lines have exceeded the sugar content of field-grown sugarcane. The massive increase of sugar accumulation in sorghum would boost its biofuel production at the commercial scale. More importantly, the results illustrated that the phenotype of high sugar content is inheritable and reliable. Additionally, the high sugar accumulation did not show any deleterious effect on plant growth morphologically in the elite engineered lines. These results demonstrate that genetic engineering on sorghum has considerable potential for biofuel and bio-industry.

## Materials and methods

### Constructs of sucrose isomerase gene

Constructs were prepared by recombining four parts. The first part is a 1.2 Kb sugarcane *ScR1MYB1 A1* promoter (GenBank EU719199) [31] or a sugarcane *loading stem gene* promoter (*LSG2,* GeneBank JQ920356) [35]. The second part is a fragment encoding signal peptide of sweet potato sporamin NTPP as described in the reports [30, 38]. The third part is a modified gene version (GenBank KC147726) encoding the UQ68J *SI* enzyme [24, 31]. The fourth part is a terminator complex including three contiguous plant transcriptional terminator regions which are intended to block read-through transcription in either direction [31] (Additional file 1: Fig. S2).

### Sorghum transformation

Sweet sorghum has been considered as one of the most recalcitrant crops in terms of genetic transformation [33]. To successfully introduce the engineered *SI* construct into the large biomass sweet sorghum lines, an inbred line of grain sorghum T×430 was first transformed. Then the T×430 transgenic lines were used as male parents for crossing with elite sweet sorghum cultivars Rio, and R9188 as female parents. Rio and R9188 are advantageous for large biomass and have been used as male-sterile parents in sorghum breeding projects. R9188 is shorter than Rio.

Plasmids containing the sucrose isomerase gene driven either by *LSG2* promoter or *ScR1MYB1 A1* promoter, are co-precipitated on gold particles with *pUKN* construct [39, 49]. Transformation protocol by particle bombardment, selection of transgenic lines, plant regeneration, and growth conditions in the glasshouse were described as GQ Liu, BC Campbell and ID Godwin [49]. Briefly, embryogenic calli derived from immature embryos (11–15-day post-anthesis) were used as explants for transformation. Transformed calli were cultured for 8–12 weeks on selective regeneration media containing 30 mg L^−1^ geneticin with subculturing fortnightly. Putative transgenic shoots were subsequently subcultured onto selective rooting media for 4 weeks following by 3-day acclimation. Details of the sorghum tissue culture were as described by GQ Liu, EK Gilding and ID Godwin [50].

### PCR screening

Genomic DNA was extracted from the young leaves of the transgenic and non-transgenic plantlets prior to potting out in the glasshouse. Extracted DNA quality and concentration were determined using a NanoDrop 2000 spectrophotometer (Thermo Scientific). To confirm the *sucrose isomerase* (*SI*) gene, specific primers were designed (Forward: 5′-AGCAACCCGATCTCAACTGG-3′ and Reverse: 5′-ACGGAGTCGTTCCATTGCAT-3′). PCR screening was undertaken in 20 μl reaction each containing 20 ng of template DNA, 0.5 μM of each specific primer and 10 μl of Taq 2 × Master Mix (New England BioLabs). PCR reactions were performed using a BIO-RAD T100 Thermal Cycler®. The PCR program comprised of an initial denaturation at 95 °C for 7 min, followed by 35 amplification cycles consisting of; 95 °C for 30 s, 60 °C for 30 s, 72 °C for 1 min, and a final elongation step of 72 °C for 7 min. PCR products were separated by 1.0% gel electrophoresis at 120 V for 1.5 h (Additional file 1: Fig. S3).

### Growth conditions and crossing

Following the hardening off period, *SI-*positive transgenic plantlets and negative controls were transferred to 20-L pots with three plantlets. Pots were randomized and grown in a temperature-controlled glasshouse (18–28 °C) for around 95 days until physiological maturity. Generally, transgenic plants and the controls started flowering 60 days after moving into the glasshouse. The transgenic plants grew as healthily as the control plants in morphology (Additional file 1: Fig. S1). Sweet sorghum seeds of Rio, and R9188 were sowed in the same glasshouse in different batches with 2-week interval to meet the flowering time of the elite transgenic lines for crossing. The crossing was performed as described [51].

### Measuring sugar content by high-performance liquid chromatography electrochemical detection (HPLC-ED)

For stalk samples, a transverse tissue slice was taken at the mid-point of each designated internode and cut into radial sectors that were proportionately representative of the different stalk tissues. Sectors were placed on a support screen (Promega Spin Basket, Madison, WI) within a 1.5-mL microfuge tube. Samples were frozen by liquid nitrogen for 20 min, and then were thawed on ice and centrifuged at 10 000 g for 15 min at 4 °C to collect the stem juice. After that, juice was boiled for 5 min to inactivate enzymes. The insoluble material was removed by centrifugation at 16 000 g for 20 min at 4 °C. The comparative tests were conducted on internodes before. Sugar concentrations, measured from this procedure, are equivalent to the sugar content from the juice which was crushed manually. Moreover, this procedure was adaptable to large scale samples. FWs were recorded before and after juice extraction and residual dry weights (DWs) were measured after 72 h at 75 °C for tissues, or 90 °C for juice samples. Water contents were measured as well.

The resolution and quantification of isomaltulose, trehalulose, sucrose, glucose and fructose were achieved by isocratic HPLC at high pH (120 mM NaOH), using a Dionex BioLC system (Sunnyvale, CA) with PA20 analytical anion exchange column and quad waveform pulsed ED, with calibration against a dilution series of sugar standards for every sample batch [15, 38]. Sugar concentrations were calibrated in the procedure and presented as sucrose equivalents in juice. Total sugar contents were calculated on an FW and DW basis, taking account of the residual juice in internode tissues after centrifugation (up to 60% of total juice) and assuming 10% reduction in solute concentration in residual juice relative to first extracted juice [52]. For leaf samples, about 1 g FW of leaf without midrib was taken at one-third of the distance from the dewlap to the leaf tip. For root samples, about 0.5 g FW of young roots was taken from the interface between the soil and pot. Fluids were extracted and assayed by the same freeze–thaw-centrifuge-HPLC method described above for stalk samples.

### qRT-PCR of the ***SI*** gene expression in T_1_ generation

The T_1_ progeny fresh leaf samples of A5, L2, L9, and T×430 (the wild-type control) were collected from the glasshouse. Leaf samples were ground using liquid nitrogen. The DNA extraction kit (ISOLATE II Plant DNA Kit, BIOLINE Cat No. Bio-52070) was used and the protocol was utilized to obtain total genomic DNA for identifying positive progenies of *NPT*II and *SI* genes. The RNA extraction kit (ISOLATE II RNA Mini Kit, BIOLINE BIO-52072) was utilized to obtain total RNA from the fourth internode of positive T_1_ plants 20-day post-anthesis. For real-time PCR, the total RNA was transcripted into cDNAs (GoScript™ Reverse Transcription, Promega, REF A5001). Then the GoTaq 1-Step RT-qPCR (Promega REF A6021) was deployed in the Bio-RAD CFX96™ Real-Time System C1000 Touch™ Thermal cycler. RT-PCR SI primers (SI-forward: CGACATCAGCGACTACAGGA; SI-reverse: CCTTGGAAGATGAACGGTGT) were used to quantify the amount of *SI* transcript, which was expressed relative to the reference gene sorghum elongation factor 1-alpha (Sb02g036420; amplification of the reference gene using primers REF-forward: CCCAAGTACTCCAAGGCTCG and REF-reverse: ATGTTGTCACCCTCGAACCC). The data was analysed using LinReg-PCR (Ramakers et al. 2003). Three biological replicates of each sample were used for RT-PCR.

### Gas exchange and chlorophyll fluorescence measurements

The photosynthetic electron transport rate was estimated from the fluorescence light curve generated using a fiber-optic MINI-PAM/F (Heinz Waltz GmbH, Effeltrich, Germany) and leaf-clip holder 2030B positioned at one-tenth of the distance from the dewlap to the leaf tip. The MINI-PAM light intensity, saturation pulse intensity, saturation pulse width, leaf absorption factor and illumination time were set at 680 µmol/m2/s, 680 µmol/m2/s, 0.8 s, 0.84 and 10 s, respectively. The internal temperature of the MINI-PAM was controlled between 25 and 30 °C during measurement. An LI-6400 portable photosynthesis system (LI-COR, Lincoln, NE, USA) was used to measure CO_2_ fixation rates on the same leaves. Measurements were performed on more than three replicate plants per progeny.

### Plasmalemma vesicle (PMV) isolation and transport assays

The blades of the second and third leaves from the top without midribs (12.5 g FW) were homogenized in 50 mL solution which contains 240 mM sorbitol, 50 mM N-2-hydroxyethylpiperazine-N’ 2-ethanesulphonic acid (HEPES), 3 mM ethyleneglycol-bis (βaminoethylether)-*N*, *N*’-tetraacetic acid (EGTA), 3 mM dithiothreitol (DTT), 10 mM KCl, 0.5% bovine serum albumin (BSA), 0.6% polyvinylpyrrolidone (PVP) and 2 mM phenylmethyl sulphonyl fluoride (PMSF) (adjusted to pH 8.0 using solid Bistris propane) at 4 °C. The homogenate was filtered through four layers of cheesecloth to remove tissue debris and then centrifuged at 10,000 g for 10 min to remove mitochondria and chloroplasts. Microsomal membranes were pelleted by centrifugation at 50 000 g for 60 min. PMVs were purified from the microsomal fraction by phase partitioning [36], washed in 25 mL of sorbitol-based re-suspension buffer (SBRB) (330 mM sorbitol, 2 mM HEPES, 0.1 mM DTT, 10 mM KCl, pH 8.0 with solid Bistris propane), repelleted by centrifugation at 50,000 g for 60 min and resuspended at 3–5 mg FW mL^−1^ of re-suspension buffer. The phase-purified PMVs were layered over a 20 to 50% sucrose gradient in 2 mM HEPES, 1 mM HCl and 1 mM DTT (pH 8.0 with solid Bistris propane), centrifuged at 100 000 g for 15 h and collected in 1-mL fractions. The fractions were washed in 11 mL SBRB and pelleted by centrifugation at 100 000 g for 60 min. The pellet was suspended in 0.4 mL of SBRB. The purity of samples was checked using routine tests for enzymatic activities of other cellular membrane types.

Transport assays were conducted at 12 °C using three replicate reactions per treatment (Bush et al., 1996). Briefly, for each reaction mixture, 20 µL of resuspended PMVs were diluted into 400 µL of assay buffer (as for SBRB, except adjusted to pH 6.0 with solid 2 [*N*-morpholino ethane sulphonic acid (MES)] containing 0.2 µCi (^14^C) sucrose and unlabelled sucrose to the desired concentration. At each time point, vesicles from one reaction mixture were collected on 0.45-µm filters and rinsed three times with 0.6 mL of assay buffer containing only unlabelled sucrose (1 mM). The accumulated radioactivity was measured by scintillation spectrometry. The difference between samples with and without 5 µM carbonyl cyanide *m*-chlorophenyl hydrazone (CCCP) was defined as *∆*pH-dependent sucrose transport.

### Internode tissue fractionation and enzyme assays

Transverse sections of each internode were divided into the outer rind of 2 mm thickness and two internal concentric cylinders at equal distances along the stalk radius. Of these, the central parenchyma-rich zone and the peripheral vascular-rich zone were examined for invertase activity. Furthermore, vascular bundles were separated by dissection from parenchyma tissue in the central zone for separate assays. The separated tissues were frozen immediately in liquid nitrogen for enzyme extraction, followed by the determination of CWI activity. Three replicate plants or dissected tissue subsamples were used for each assay [53].

The SI enzyme was extracted by grinding the frozen cells in a chilled mortar using three volumes of extraction buffer that contained 0.1 M Hepes–KOH buffer(pH7.5), 10 mM MgCl_2_, 2 mM EDTA, 2 mM EGTA, 10% glycerol, 5 mM DTT, 2% polyvinylpolypyrrolidone and 1 × complete protease inhibitor (Roche, Mannheim, Germany). The homogenate was immediately centrifuged at 10 000 g for 15 min at 4 °C. The supernatant was immediately desalted on a PD-10 column (GE Healthcare, Buckinghamshire, UK) that was pre-equilibrated and eluted using the extraction buffer. Protein concentration was assayed by the Bradford reaction using a Bio-Rad kit (Hercules, CA, USA) with bovine serum albumin standards. *SI* activity was measured by incubating enzyme extract with 292 mM sucrose solution in 0.1 M citrate–phosphate buffer (pH 6.0) at 30 °C.

## Supplementary Information

**Additional file 1**: **Fig. S1**. Transgenic sorghum lines were grown in a PC2 glasshouse. (a) 1 week; (b) 2 weeks; (c) 3 weeks; (d) 5 weeks; (e) 7 weeks; (f) 10 weeks in the glasshouse; (g) mature T×430; (h) mature A5; (f) mature L9. **Fig. S2**. The Construct Used for Gene Transformation. Stem-specific Promoter: either *A1* resulting in high gene expression in the mature stalk or *LSG2* resulting in high gene expression in the loading sucrose section of the stalk. Vacuole leading: encoding a propeptide to guide *SI* gene products to vacuole, where sucrose accumulates. Non-silence target gene: sucrose isomerase without motifs gene silencing in plants. Multiple terminators: three recombined terminators complex to guarantee the proper termination of gene transcription. **Fig. S3**. PCR screening of sucrose isomerase gene in transgenic *LSG2* lines. The agarose gel displayed PCR results of transgenic lines. M: DNA ladder; NC: negative control; Transgenic lines L1, L2, L3, L4, L5, L7, L9, L14, and L16 were positive of the sucrose isomerase gene; Transgenic lines L6, L8, L10, L11, L12, L13,L15, and L17 were negative of the sucrose isomerase gene. **Fig. S4**. Sugar profile of T_1_ L9 lines. Sugars, including isomaltulose, were measured 20-day post-anthesis in the middle section of internode 4 (counted from top). L9 is one of the top lines in T_0_ transgenic lines. L9-2, 9-3, 9-6, 9-7, 9-11, 9-12 are isomaltulose positive samples. Nil-L9-4, 9-8 and 9-9 are null-segregant samples in T_1_ generation. T×430-1, T×430-2, and T×430-3 are the non-transformed control. **Fig. S5**. PCR screening of sucrose isomerase gene in T_1_ lines. The agarose gel displayed PCR results of transgenic lines and controls. M: 1 Kb DNA ladder; Positive A5 T_1_ progenies: A5-2 and A5-4; Positive L2 T_1_ progenies: L2-1, L2-3 and L2-6; Positive L9 T_1_ progenies: L9-2, and L9-4; T×430-1, T×430-2 are non-transgenic control samples; PC: positive plasmid control (*LSG2*). **Table S1**. Sugar profile of controls and positive transgenic lines with isomaltulose. **Table S2**. Sugar profile of F_1_ hybrid lines of R9188 X L9.

## Data Availability

The datasets supporting the conclusions of this article are included in the article and its Additional files.

## Abbreviations

SI: Sucrose isomerase
FW: Fresh weight
NTPP: N-terminal pro-peptide
PCR: Polymerase chain reaction
qRT-PCR: Quantitative real-time PCR
HPLC: High-performance liquid chromatography
PAR: Photosynthetically active radiation
PMV: Plasma membrane vesicles
CWI: Cell wall invertase
FT: Fructosyltransferase
HPLC-ED: High-performance liquid chromatography electrochemical detection
DW: Dry weight
PMV: Plasmalemma vesicle
HEPES: N-2-hydroxyethylpiperazine-N’ 2-ethanesulphonic acid
EGTA: Ethyleneglycol-bis (βaminoethylether)-N, N’-tetraacetic acid
DTT: Dithiothreitol
BSA: Bovine serum albumin
PVP: Polyvinylpyrrolidone
PMSF: Phenylmethyl sulphonyl fluoride
SBRB: Sorbitol-based re-suspension buffer
MES: N-morpholino ethane sulphonic acid
CCCP: Carbonyl cyanide m-chlorophenyl hydrazone
