# Supplementary material for: Stem vacuole-targetted sucrose isomerase enhances sugar content in sorghum
Source: Biotechnol Biofuels. 2021 Mar 1;14:53. doi: 10.1186/s13068-021-01907-z (PMC7923521; doi:10.1186/s13068-021-01907-z)
Supplement: Supplementary file 1 — Additional file 1: Fig. S1. Transgenic sorghum lines were grown in a PC2 glasshouse. (a) 1 week; (b) 2 weeks; (c) 3 weeks; (d) 5 weeks; (e) 7 weeks; (f) 10 weeks in the glasshouse; (g) mature T×430; (h) mature A5; (f) mature L9. Fig. S2. The Construct Used for Gene Transformation. Stem-specific Promoter: either A1 resulting in high gene expression in the mature stalk or LSG2 resulting in high gene expression in the loading sucrose section of the stalk. Vacuole leading: encoding a propeptide to guide SI gene products to vacuole, where sucrose accumulates. Non-silence target gene: sucrose isomerase without motifs gene silencing in plants. Multiple terminators: three recombined terminators complex to guarantee the proper termination of gene transcription. Fig. S3. PCR screening of sucrose isomerase gene in transgenic LSG2 lines. The agarose gel displayed PCR results of transgenic lines. M: DNA ladder; NC: negative control; Transgenic lines L1, L2, L3, L4, L5, L7, L9, L14, and L16 were positive of the sucrose isomerase gene; Transgenic lines L6, L8, L10, L11, L12, L13,L15, and L17 were negative of the sucrose isomerase gene. Fig. S4. Sugar profile of T1 L9 lines. Sugars, including isomaltulose, were measured 20-day post-anthesis in the middle section of internode 4 (counted from top). L9 is one of the top lines in T0 transgenic lines. L9-2, 9-3, 9-6, 9-7, 9-11, 9-12 are isomaltulose positive samples. Nil-L9-4, 9-8 and 9-9 are null-segregant samples in T1 generation. T×430-1, T×430-2, and T×430-3 are the non-transformed control. Fig. S5. PCR screening of sucrose isomerase gene in T1 lines. The agarose gel displayed PCR results of transgenic lines and controls. M: 1 Kb DNA ladder; Positive A5 T1 progenies: A5-2 and A5-4; Positive L2 T1 progenies: L2-1, L2-3 and L2-6; Positive L9 T1 progenies: L9-2, and L9-4; T×430-1, T×430-2 are non-transgenic control samples; PC: positive plasmid control (LSG2). Table S1. Sugar profile of controls and positive transgenic lines with isoma [file 13068_2021_1907_MOESM1_ESM.docx]

**Supplementary materials**


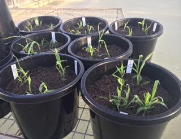

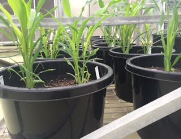

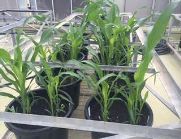

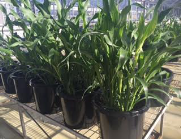

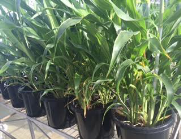

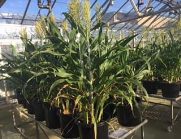


a

b

c

d

e

f


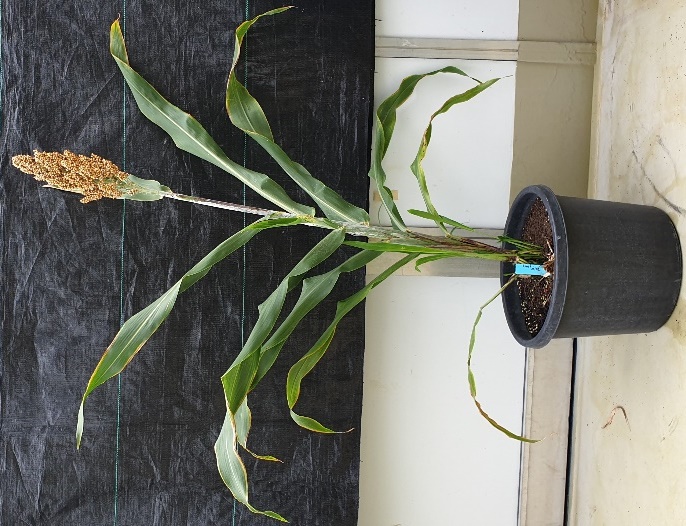

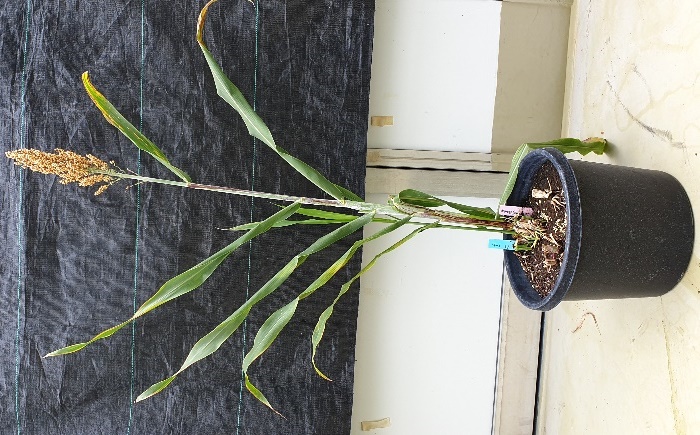

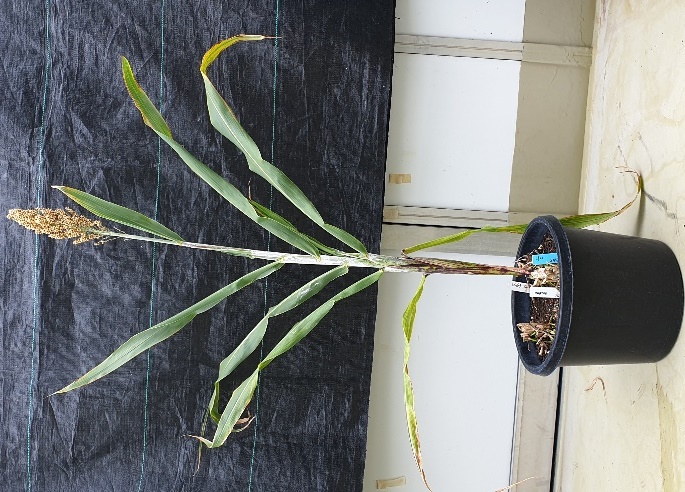


i

h

g

**Fig. S1. Transgenic sorghum lines were grown in a PC2 glasshouse.** (**a**) one week; (**b**) two weeks; (**c**) three weeks; (**d**) five weeks; (**e**) seven weeks; (**f**) ten weeks in the glasshouse; (**g**) mature Tx430; (**h**) mature A5; (**f**) mature L9.


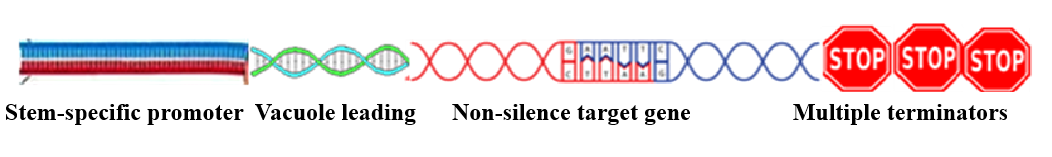


**Fig. S2. The Construct Used for Gene Transformation.** Stem-specific Promoter: either *A1* resulting in high gene expression in the mature stalk or *LSG2* resulting in high gene expression in the loading sucrose section of the stalk. Vacuole leading: encoding a propeptide to guide *SI* gene products to vacuole where sucrose accumulates. Non-silence target gene: *sucrose isomerase* without motifs gene silencing in plants. Multiple terminators: three recombined terminators complex to guarantee the proper termination of gene transcription.


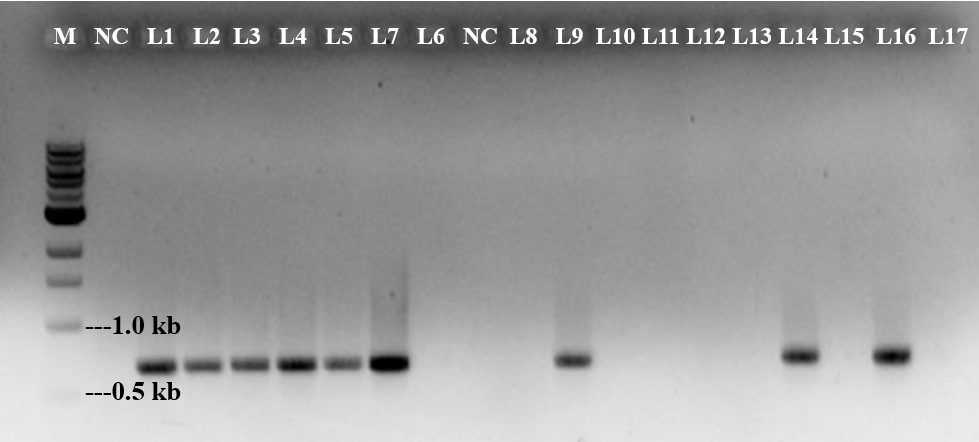


**Fig. S3 PCR screening of sucrose isomerase gene in transgenic *LSG2* lines.** The agarose gel displayed PCR results of transgenic lines. M: DNA ladder; NC: negative control; Transgenic lines L1, L2, L3, L4, L5, L7, L9, L14, and L16 were positive of the sucrose isomerase gene; Transgenic lines L6, L8, L10, L11, L12, L13,L15, and L17 were negative of the sucrose isomerase gene.


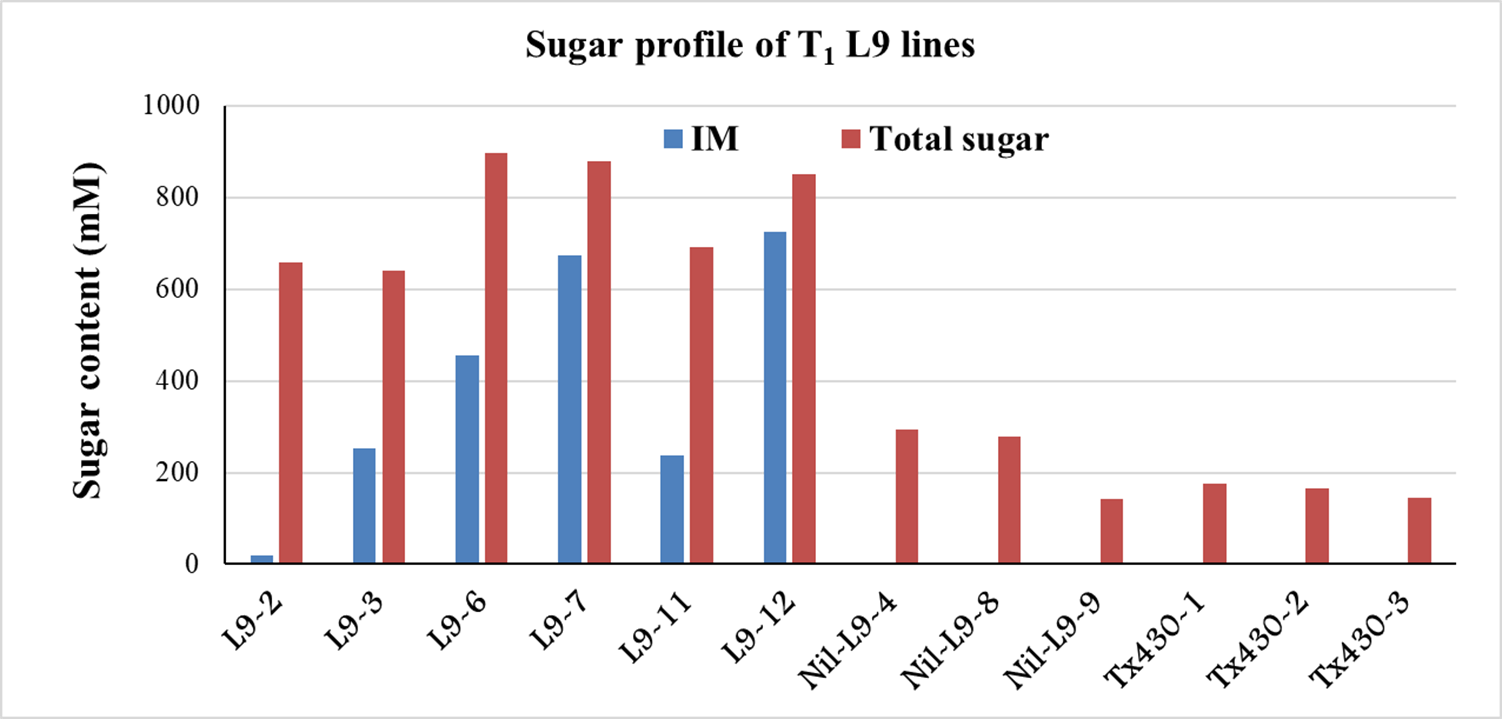


**Fig. S4 Sugar profile of T_1_ L9 lines.** Sugars, including isomaltulose, were measured 20 days post-anthesis in the middle section of internode 4 (counted from top). L9 is one of the top lines in T_0_ transgenic lines. L9-2, 9-3, 9-6, 9-7, 9-11, 9-12 are isomaltulose positive samples. Nil-L9-4, 9-8 and 9-9 are null-segregant samples in T_1_ generation. Tx430-1, Tx430-2, and Tx430-3 are the non-transformed control.


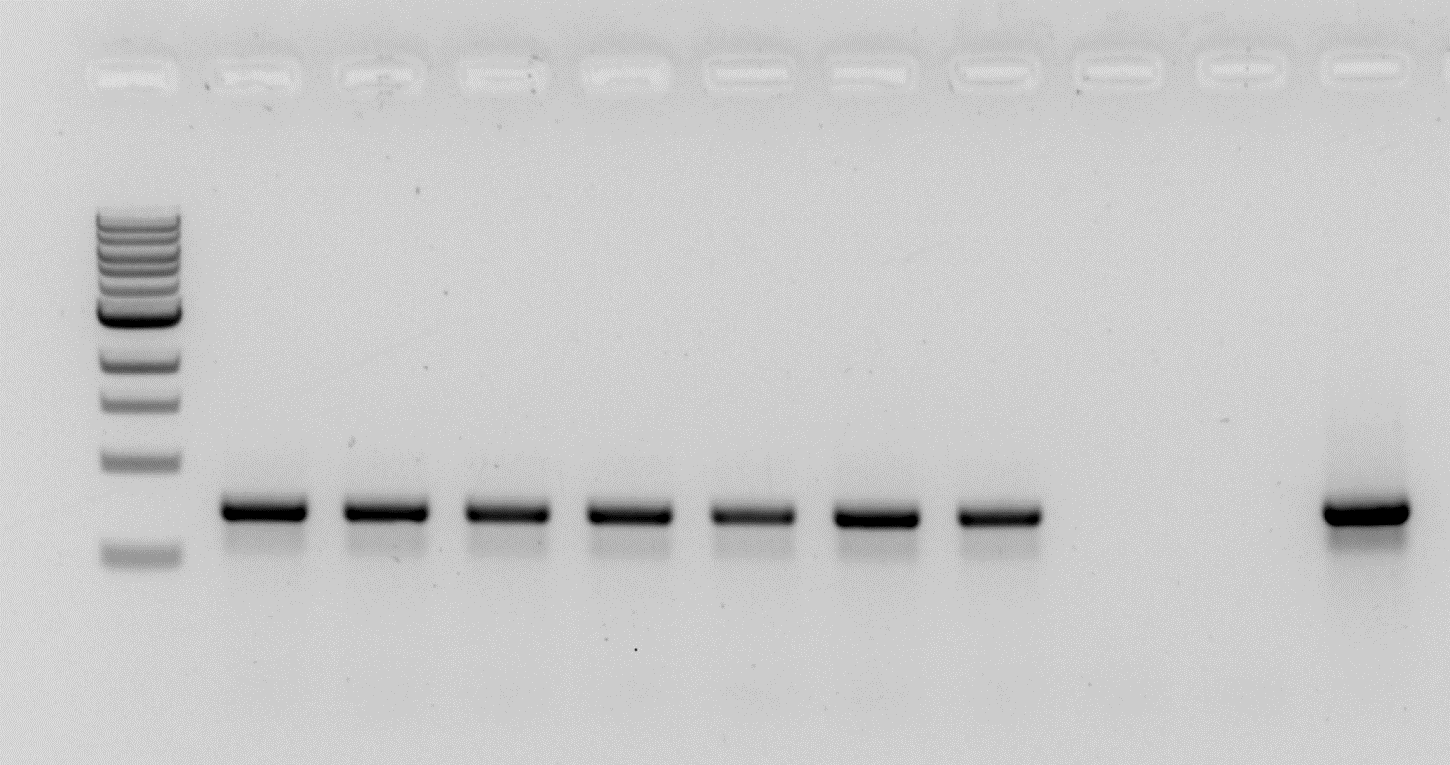


**---1.0kb**

**---0.5kb**

**M A5-2 A5-4 L2-1 L2-3 L2-6 L9-2 L9-4 Tx-1 Tx-2 PC**

**Fig. S5 PCR screening of sucrose isomerase gene in T_1_ lines.** The agarose gel displayed PCR results of transgenic lines and controls. M: 1 Kb DNA ladder; Positive A5 T_1_ progenies: A5-2 and A5-4; Positive L2 T_1_ progenies: L2-1, L2-3 and L2-6; Positive L9 T_1_ progenies: L9-2, and L9-4; Tx430-1, Tx430-2 are non-transgenic control samples; PC: positive plasmid control (*LSG2*).

**Table S1 Sugar profile of controls and positive transgenic lines with isomaltulose**

| Sample*  Name | Glucose  (µM) | Fructose  (µM) | Sucrose  (µM) | Trehalulose  (µM) | Isomaltulose  (µM) | Total sugar**  (µM) |
| --- | --- | --- | --- | --- | --- | --- |
| T1 | 1.27 | 1.41 | 8.03 | n.a. | n.a. | 9.37 |
| T2 | 3.11 | 1.29 | 9.20 | n.a. | n.a. | 11.4 |
| T3 | 5.48 | 5.81 | 63.86 | n.a. | n.a. | 69.50 |
| T4 | 33.52 | 32.90 | 70.19 | n.a. | n.a. | 103.4 |
| T5 | 55.59 | 56.97 | 60.22 | n.a. | n.a. | 116.5 |
| A131 | 71.91 | 61.08 | 578.17 | n.a. | 4.39 | 649.06 |
| A13 | 32.61 | 23.29 | 498.41 | n.a. | 9.76 | 536.12 |
| A11 | 40.92 | 8.07 | 195.74 | 2.54 | 40.82 | 263.60 |
| A15 | 55.60 | 4.79 | 393.47 | 4.95 | 141.24 | 569.86 |
| A5 | 17.37 | 34.66 | 382.09 | 14.63 | 329.06 | 751.80 |
| A52 | 7.25 | 25.25 | 399.62 | 61.94 | 361.01 | 838.82 |
| A51 | 8.38 | 17.64 | 527.30 | 26.15 | 446.34 | 1012.8 |
| L1 | 67.93 | 64.19 | 121.36 | n.a. | 15.06 | 202.48 |
| L2 | 1.52 | 0.78 | 14.45 | n.a. | 28.16 | 43.76 |
| L24 | 1.21 | 0.80 | 14.75 | n.a. | 42.53 | 58.285 |
| L14 | 45.24 | 46.96 | 475.61 | 1.97 | 56.13 | 579.81 |
| L19 | 48.36 | 37.08 | 600.14 | 10.14 | 95.35 | 748.35 |
| L3 | 39.08 | 34.50 | 629.87 | 8.80 | 130.36 | 805.82 |
| L9 | 4.81 | 8.92 | 482.03 | 15.87 | 328.65 | 833.415 |
| L16 | 52.45 | 62.59 | 295.34 | 18.01 | 384.80 | 755.67 |
| L7 | 25.27 | 46.28 | 178.98 | 21.44 | 432.51 | 668.705 |

*: From T1 to T5 are samples of wildtype control Tx430. A and L lines are transgenic lines with different promoters.

**: Total sugar content = ½ (Glucose + Fructose) + Sucrose + Trehalulose + Isomaltulose

**Table S2 Sugar profile of F_1_ hybrid lines of L9 X R9188 (L9R9)**

| Sample* | PCR | | Glucose (µM) | Fructose (µM) | Sucrose (µM) | Trehalulose (µM) | Isomaltulose (µM) | Total sugar** (µM) |
| --- | --- | --- | --- | --- | --- | --- | --- | --- |
|  | NPTII | SI |  |  |  |  |  |  |
| Tx430-1 | N | N | 120.09 | 31.98 | n.a. | n.a. | n.a. | 76.04 |
| Tx430-2 | N | N | 153.95 | 6.41 | n.a. | n.a. | n.a. | 80.18 |
| Tx430-3 | N | N | 70.18 | 24.52 | 75.84 | n.a. | n.a. | 123.19 |
| **Average** |  |  | **114.74** | **20.97** | **75.84** |  |  | **93.14** |
|  |  |  |  |  |  |  |  |  |
| R9188-1 | N | N | 5.71 | 1.42 | 193.80 | n.a. | n.a. | 197.36 |
| R9188-2 | N | N | 17.89 | 1.04 | 271.23 | n.a. | n.a. | 280.70 |
| R9188-3 | N | N | 8.87 | 0.58 | 300.27 | n.a. | n.a. | 304.99 |
| Average |  |  | **10.82** | **1.01** | **255.10** |  |  | **261.02** |
|  |  |  |  |  |  |  |  |  |
| L9R9-4 | N | N | 135.41 | 13.48 | 290.63 | n.a. | n.a. | 365.07 |
| L9R9-6 | N | N | 75.63 | 41.06 | 410.39 | n.a. | n.a. | 468.74 |
| L9R9-8 | N | N | 39.91 | 15.62 | 248.98 | n.a. | n.a. | 276.75 |
| L9R9-10 | N | N | 101.85 | 24.87 | 207.33 | n.a. | n.a. | 270.69 |
| L9R9-12 | N | N | 65.78 | 3.70 | 60.62 | n.a. | n.a. | 95.36 |
| L9R9-13 | N | N | 83.22 | 10.38 | 263.04 | n.a. | n.a. | 309.84 |
| L9R9-15 | N | N | 43.39 | 7.99 | 330.86 | n.a. | n.a. | 356.55 |
| L9R9-16 | N | N | 122.48 | 16.62 | 100.38 | n.a. | n.a. | 169.93 |
| L9R9-25 | N | N | 72.87 | 15.34 | 510.48 | n.a. | n.a. | 554.58 |
| L9R9-22 | N | N | 172.83 | 26.73 | 273.94 | n.a. | n.a. | 373.72 |
| L9R9-23 | N | N | 53.80 | 8.78 | 444.67 | n.a. | n.a. | 475.96 |
| L9R9-24 | N | N | 22.85 | 2.08 | 579.60 | n.a. | n.a. | 592.07 |
| L9R9-29 | N | N | 19.00 | 1.90 | 300.00 | n.a. | n.a. | 310.45 |
| L9R9-28 | N | N | 7.00 | 5.00 | 175.00 | n.a. | n.a. | 181.00 |
| **Average N** |  |  | **72.57** | **13.83** | **299.71** |  |  | **342.91** |
|  |  |  |  |  |  |  |  |  |
| L9R9-5 | P | P | 42.32 | 26.51 | 439.68 | n.a. | 2.13 | 476.22 |
| L9R9-26 | P | P | 23.53 | 23.44 | 539.17 | n.a. | 2.51 | 565.16 |
| L9R9-21 | P | P | 119.84 | 18.42 | n.a. | n.a. | 2.89 | 72.02 |
| L9R9-1 | P | P | 22.96 | 5.75 | 157.64 | 14.85 | 307.87 | 494.72 |
| L9R9-3 | P | P | 42.62 | 5.82 | 59.83 | 19.12 | 401.57 | 504.74 |
| L9R9-2 | P | P | 5.40 | 3.67 | 172.68 | 19.35 | 406.87 | 603.44 |
| L9R9-27 | P | P | 32.05 | 4.77 | n.a. | 22.81 | 412.37 | 453.59 |
| L9R9-7 | P | P | 22.67 | 12.67 | 82.91 | 38.08 | 514.21 | 652.88 |
| L9R9-19 | P | P | 38.85 | 4.38 | 82.10 | 25.91 | 526.75 | 656.37 |
| L9R9-20 | P | P | 18.86 | 1.11 | 164.94 | 45.85 | 543.08 | 763.86 |
| L9R9-17 | P | P | 40.65 | 5.60 | 4.42 | 32.35 | 547.95 | 607.85 |
| L9R9-9 | P | P | 3.36 | 3.83 | n.a. | 33.17 | 565.38 | 602.14 |
| **Average P** |  |  | **34.43** | **9.66** | **189.26** | **27.95** | **352.80** | **537.75** |

*: Wildtype controls Tx430, and R9188 are measured in triplicates. Samples are PCR screened for NPTII and surose isomerase (SI) genes.

**: Total sugar content = ½ (Glucose + Fructose) + Sucrose + Trehalulose + Isomaltulose
